# Publisher Correction: Spin-Peierls instability of the U(1) Dirac spin liquid

**DOI:** 10.1038/s41467-024-54023-5

**Published:** 2024-11-06

**Authors:** Urban F. P. Seifert, Josef Willsher, Markus Drescher, Frank Pollmann, Johannes Knolle

**Affiliations:** 1 Kavli Institute for Theoretical Physics, University of California, Santa Barbara, CA USA; 2 Technical University of Munich, TUM School of Natural Sciences, Physics Department, Garching, Germany; 3 Munich Center for Quantum Science and Technology (MCQST), Schellingstr. 4, München, Germany; 4 Blackett Laboratory, Imperial College London, London, United Kingdom; 5 Present Address: Institute for Theoretical Physics, University of Cologne, Cologne, Germany

**Keywords:** Magnetic properties and materials, Quantum fluids and solids

Correction to: *Nature Communications* 10.1038/s41467-024-51367-w, published online 19 August 2024

In this article the following paragraph was duplicated, ‘First, we derive the phase diagram by evaluating the correction to the Xa phonon energy ω2 a via Eq. (15). For temperatures T ≫ ω, scaling arguments imply that to leading order χ0 aðω,TÞ ∼ Tð32ΔΦÞ c + Oðω = TÞ with some constant c 42, and from (15) it follows that the phonon frequency (pole of Ga(ω)) is shifted downwards.’ The original article has now been updated.

