# Supplementary material for: Spin-Peierls instability of the U(1) Dirac spin liquid
Source: Nat Commun. 2024 Aug 19;15:7110. doi: 10.1038/s41467-024-51367-w (PMC11333588; doi:10.1038/s41467-024-51367-w)
Supplement: Supplementary file 1 — Supplementary Information [file 41467_2024_51367_MOESM1_ESM.pdf]

Supplementary Information:
Spin-Peierls instability of the U(1) Dirac spin liquid

Urban F. P. Seifert[1,*], Josef Willsher[2,3,*], Markus Drescher[2,3], Frank Pollmann[2,3], and Johannes Knolle[2,3,4]

[1] *Kavli Institute for Theoretical Physics, University of California, Santa Barbara, CA 93106*
[2] *Technical University of Munich, TUM School of Natural Sciences, Physics Department, 85748 Garching, Germany*
[3] *Munich Center for Quantum Science and Technology (MCQST), Schellingstr. 4, 80799 München, Germany*
[4] *Blackett Laboratory, Imperial College London, London SW7 2AZ, United Kingdom*
[*] *These authors contributed equally*

Supplementary Note 1: Effective action for distortion modes

Mass terms and mass-monopole OPE

In the main text, we have focussed on a coupling of lattice distortion modes to monopole operators of the QED_3 CFT, which are expected to be the most relevant operators in the theory. The next lowest-lying operators are the adjoint fermion masses

$$M^{\alpha\beta} = \overline{\psi}\sigma^\alpha\tau^\beta\psi \tag{1}$$

with $\alpha, \beta = 0, 1, 2, 3$ and σ/τ act on spin/valley indices of the fermions. The masses $M^{\alpha\beta}$ transform in a sixteen-dimensional reducible representation of SU(4), which splits into the singlet and adjoint irreducible representations $\mathbf{16} = \mathbf{1} \oplus \mathbf{15}$. The scaling dimension of the adjoint mass term $M^{\mu\nu}$ (with $\mu = \nu = 0$ excluded) is strongly relevant, with $\Delta_M = 1.46$ in the large-N_f expansion [1] and $\Delta_M \in (1.33, 1.66)$ according to a recent bootstrap study [2]. The singlet M_{00} corresponds to a chiral mass term and can be tuned to produce a chiral spin-liquid state. In large-N_f, this term is irrelevant $\Delta_0 = 3.08$, and thus it is believed that spontaneous chiral symmetry breaking does not occur [3, 4, 5].

The operator product expansion (OPE) states how operators approaching each other are expanded in the basis of primaries as

$$\lim_{x\to y} \mathcal{O}_i(x)\mathcal{O}_j(y) = \lim_{x\to y} \sum_k \frac{C_{ij}^k}{|x-y|^{\Delta_i+\Delta_j-\Delta_k}} \mathcal{O}_k(y), \tag{2}$$

where the C_{ij}^k are the OPE coefficients. If the CFT is endowed with some global symmetry group $\mathcal{G}$, each term on the RHS of (2) will transform in some irreducible representation of $\mathcal{G} \times \mathcal{G}$. We stress that Supplementary Eq. (2) corresponds to the asymptotic formulation of the OPE, but in general, OPEs can be shown to be convergent series if all primaries and their descendants are included. The full asymptotic form of the operators can be written in terms of the two real OPE coefficents $C_{\Phi\Phi}^M$ and $C_{M\Phi}^\Phi$ in the following way:

$$\Phi_a^\dagger(x)\Phi_b(y) \sim \frac{\delta_{ab}}{|x-y|^{2\Delta_\Phi}} + \frac{\mathrm{i}C_{\Phi\Phi}^M}{|x-y|^{2\Delta_\Phi-\Delta_M}} \mathcal{F}_{\mu\nu}^{ab} M^{\mu\nu}(y) + \dots \tag{3a}$$

$$\Phi_a(x)M^{\mu\nu}(y) \sim \frac{\mathrm{i}C_{\Phi M}^\Phi}{|x-y|^{\Delta_M}} \bar{\mathcal{F}}_{ab}^{\mu\nu} \Phi_b(y) + \dots, \tag{3b}$$

where the tensor $\mathcal{F}_{\mu\nu}^{ab}$ maps elements of the adjoint-SU(4) representation to rank-2 antisymmetric representations of SO(6) and $\bar{\mathcal{F}}_{ab}^{\mu\nu}$ its inverse. Implicit sums over (μ, ν) are taken to exclude the $(0, 0)$ element. The nonzero components are $\mathcal{F}_{0i}^{ab} = \epsilon^{abi}$ for $a, b \le 3$; $\mathcal{F}_{0i}^{ab} = \epsilon^{a-3,b-3,i}$ for $a, b > 3$; and $\mathcal{F}_{ij}^{ab} = \delta_j^a\delta_{i+3}^b - \delta_{i+3}^a\delta_j^b$, as defined in Ref. [6]. Here, the components $i, j = 1, 2, 3$. We can also write the components of its inverse $\bar{\mathcal{F}}_{ab}^{\mu\nu} = \mathcal{F}_{\mu\nu}^{ab}/2$.

Effective action including fermion mass couplings

In the main text, we constructed an effective action of VBS monopoles coupled to lattice distortions by considering the leading contributions from a redefinition of the lattice $\mathbf{R}_i \to \mathbf{R}_i + \mathbf{u}_i$.

In this Supplementary Note, we will approach the task of constructing an effective theory of QED_3 coupled to a lattice distortion using a top-down approach. This path allows us to categorise couplings to all possible

	(T_1, T_2)	R	C_6	$\mathcal{T}$
M_{00}	$(+,+)$	$-$	$+$	$-$
M_{i0}	$(+,+)$	$+$	$-$	$+$
M_{01}	$(-,-)$	$+M_{03}$	$-M_{02}$	$+$
M_{02}	$(+,-)$	$-M_{02}$	$+M_{03}$	$+$
M_{03}	$(-,+)$	$+M_{01}$	$+M_{01}$	$+$
M_{i1}	$(-,-)$	$-M_{i3}$	$+M_{i2}$	$-$
M_{i2}	$(+,-)$	$+M_{i2}$	$-M_{i3}$	$-$
M_{i3}	$(-,+)$	$-M_{i1}$	$-M_{i1}$	$-$

Supplementary Table 1: Discrete symmetry transformations of fermion mass operators [7, 8].

operators in the theory which could contribute to a ground state instability. We show that the monopole indeed causes the leading instability and that the subleading operators couple to the same momentum modes of the lattice distortion.

We now write down a general set of couplings to the leading relevant operators in QED_3: the monopoles Φ_a ($a = 1 \dots 6$) and adjoint fermion masses $M_{\mu\nu}$ [$\mu, \nu = 0 \dots 3$, $(\mu,\nu) \neq (0,0)$]. The general form of this action is

$$S \sim \int \mathrm{d}^3 x \left[\sum_{a=1}^{6} (\varphi_a^* \Phi_a(x) + \text{h.c.}) + \sum_{\mu,\nu} m_{\mu\nu} M^{\mu\nu}(x) \right]. \tag{4}$$

The monopoles transform under the UV symmetries as listed in the main text, and the masses as listed in Table 1. We will preserve the lattice symmetries by constructing an appropriate object u_a that transforms in the same representation as Φ_a of the IR emergent symmetry group SU(4). Because the UV lattice symmetries embed in this emergent group, constructing an invariant SU(4)-scalar is equivalent to writing an function that is invariant under lattice symmetries.

For our physical system of interest, the question becomes whether it is possible to construct a vector u_a that transforms as the monopole under physical symmetries in Table 1 in the main text out of the physical lattice-distortion vector field $\mathbf{u}(\mathbf{r}_x)$. This object will naturally transform as a vector under the $\mathrm{SO}(3)_{\text{valley}}$ subgroup of the full SO(6), and not transform under $\mathrm{SO}(3)_{\text{spin}}$.

To classify all perturbations that could be symmetry-allowed in the presence of a deformation vector $\mathbf{u}_{\mathbf{q}}$, we must restrict our search by using the following criteria: (1) consider distortions which are periodic with wavevector $\mathbf{q}$ that is equal to that of the field theory operator and (2) are fully invariant under the discrete subgroups C_6, $\mathcal{T}$. These two requirements will be equivalent to restricting to coupling field theory operators to fields which are vectors under $\mathrm{SO}(3)_{\text{valley}}$ and trivial under $\mathrm{SO}(3)_{\text{valley}}$. Finally, by way of the correspondence between vector and adjoint representations, one can write down an object bilinear in the lattice deformation field which can couple to field theory objects in the $\mathrm{SO}(3)_{\text{valley}}$-adjoint representation.

We satisfy the above constraints by defining field u_a as

$$u_a = \mathrm{i} s_a (\mathbf{X}_a \cdot \mathbf{u}_{\mathbf{X}_a}), \tag{5}$$

where we have chosen $\mathbf{X}_a$ as a basis of three vectors separated by $2\pi/3$ angles. It satisfies translational invariance since it has the same momentum eigenvalue as the monopole, and also transforms as a vector under $\mathrm{SO}(3)_{\text{valley}}$. Under IR symmetries it transforms as the VBS monopole, as listed in Table 1 in the main text. We see it is possible to preserve the full lattice symmetry group by coupling this field to the monopoles and masses as follows:

$$\varphi_a^* = g\, u_a^*, \quad m_{0a} = \mathrm{i} m\, \mathcal{F}_{0a}^{bc} u_b^* u_c, \quad a = 1, 2, 3, \tag{6}$$

where the non-zero components give $m_{0a} = \mathrm{i} m\, \epsilon^{abc} u_b^* u_c$.

We can exclude any direct linear coupling of lattice distortions to spin-triplet monopoles Φ_a, $a = 4, 5, 6$ by spin rotation symmetry. We could instead attempt to couple distortions u_a directly to the valley-triplet fermion

masses M_{ia}, and then generate monopole couplings by using the inverse mapping between 2-dimensional and adjoint representations. However these masses also transform in the index i as a spin-vector, which could provide a hindrance to writing an invariant action. Inspecting the behavior under lattice symmetries (Table 1), we find the coupling satisfies translational invariance (1) when coupling to the (real) modes at the $\mathbf{M}_{1,2,3}$-points on the Brillouin zone edges $\tilde{\mathbf{u}}_a = \mathbf{u}_{\mathbf{M}_a}$. We are therefore motivated to write the couplings $\tilde{m}_i, \tilde{g}_i$ for $i = 1, 2, 3$,

$$m_{ia} = \tilde{m}_i \tilde{u}_a, \quad \varphi_a = -\mathrm{i}\tilde{g}_i\, \mathcal{F}^{ia}_{bc} \tilde{u}^b \tilde{u}^c, \quad a = 1, 2, 3, \tag{7}$$

with non-zero components giving $\tilde{\varphi}_{a+3} = -\mathrm{i}\tilde{g}_b \tilde{u}_a \tilde{u}_b$. However there is no choice of $\tilde{u}_a$ which leaves $m_{ia} M^{ia}$ invariant under both discrete lattice symmetries (2). For this reason, we must exclude a direct coupling between fermion masses and the $\mathbf{M}$ points from the effective action.

We are now able to write the full symmetry-allowed action at lowest order in relevant operators as a sum of VBS monopole and mass terms,

$$S_I[u_a] = \int \mathrm{d}^3 x \left[g \left(u_a^* \, \Phi_a(x) + \text{h.c.} \right) + \mathrm{i} m \, \epsilon^{abc} u_b^* u_c \, M_{0a}(x) \right], \tag{8}$$

both of which are coupled to lattice distortions via Supplementary Eq (5). There is furthermore a set of compound operators which may be added as deformations to the theory. One example is the triple-monopole term $\Phi_1 \Phi_2 \Phi_3$; allowed even on the undistorted lattice, this term is irrelevant at the conformal IR fixed point. One can equally construct double-monopole terms coupled to a commensurate lattice distortion (or appropriate combination thereof). At this point we will ignore such compound operators which have a scaling dimension $\Delta > 3/2$; this is justified by seeing that the leading IR divergence contribution to the free energy from such an operator goes as $\beta^{3-2\Delta}$ (at quadratic order in perturbation theory).

Lifting the accidental $\mathrm{U}(3)$ symmetry

The weak-coupling effective action is given as a function of the norm $|\vec{u}|$ of the complex vector $\vec{u} = (u_1, u_2, u_3)^\top$, implying invariance under global $G \in \mathrm{U}(3)$ transformations $\hat{u} \mapsto G\hat{u}$. However, this global continuous symmetry is accidental as it does not correspond to the physical symmetry group of microscopic lattice symmetries. We first note that the U(3) symmetry acts on the VBS monopoles, which transform as $\mathrm{SO}(3)_{\text{valley}}$-vectors under the emergent symmetry group of the QED_3 fixed-point action. To this end, we note that $2\mathbf{X}_1 - 2\mathbf{X}_2 = 2\mathbf{X}_2 - 2\mathbf{X}_3 = 2\mathbf{X}_1 - 2\mathbf{X}_3 = 0$ up to reciprocal lattice vectors, and hence $|\vec{u} \cdot \vec{u}|$ transforms trivially under the $\mathrm{SO}(6) \times \mathrm{U}(1)_{\text{top}}$ symmetry group. This term will generically appear in a strong-coupling effective action obtained after 'integrating out' the DSL and breaks the U(3) redundancy down to $\mathrm{SO}(3)_{\text{valley}}$.

We will now derive the subleading contribution to the perturbative free energy functional due to lattice-fermion mass couplings and recover exactly this term. Performing the same calculation as in the main text for lattice-monopole couplings, we enter the weak coupling regime $g^2 \beta^{3-2\Delta_\Phi} |\vec{u}| \ll 1$ and perform a perturbative expansion of the free energy $\langle S_m^2 \rangle / \beta V$. Using the explicitly derived structure of the coupling, we find

$$\begin{aligned} \frac{\langle S_m^2 \rangle_{\mathrm{QED}_3}}{\beta V} &= -m^2 \int \mathrm{d}^3 x \, \frac{\epsilon^{abc} \epsilon^{ade} \, u_b u_d^* \, u_c u_e^*}{|x|^{2\Delta_M}} \\ &= c_{\Delta_M} m^2 \beta^{3-2\Delta_M} \left(|\vec{u} \cdot \vec{u}|^2 - |\vec{u}|^4 \right). \end{aligned} \tag{9}$$

This is IR-divergent if the exponent $\Delta_M < 3/2$, which is compatible with large-N calculations and within the range of values suggested by the conformal bootstrap [2]. Assuming this condition is satisfied, this subleading divergent contribution will be minimised by states satisfying $\vec{u} \cdot \vec{u} = 0$.

If this condition $\Delta_M < 3/2$ is not satisfied, we would still expect the perturbative action to have only an SO(3) symmetry which is inherited from the $\mathrm{SO}(3)_{\text{valley}}$ symmetry of the effective action. This inevitably occurs at higher order in perturbation theory, where the monopole-monopole and monopole-mass terms produce additional divergent contributions proportional to $\mathcal{C}(\beta)(|\vec{u} \cdot \vec{u}|^2 - |\vec{u}|^4)$ [6]. At quadratic order there is a contribution from the $M \times M$ OPE channel above, giving $\mathcal{C}^{(2)}(\beta) = c_2 \, \beta^{3-2\Delta_M}$. At higher order there are contributions from the following channels: $\Phi^\dagger \times (M \times \Phi) \to \Phi^\dagger \times \Phi$ is asymptotically $\mathcal{C}^{(3)}(\beta) = c_3 \, \beta^{6-2\Delta_\Phi - \Delta_M}$; and the channel $(\Phi^\dagger \times \Phi) \times (\Phi^\dagger \times \Phi) \to M \times M$ contributes $\mathcal{C}^{(4)}(\beta) = c_4 \, \beta^{9-4\Delta_\Phi}$. We can constrain $c_2 \sim m^2 > 0$ and $c_4 \sim g^2 (C^M_{\Phi\Phi})^2 > 0$ to be positive, but the sign of c_3 cannot be fixed in this way. There will be multiple

additional contributions at this order (e.g. from $\langle S_g^2\rangle^2/\beta V)$ which produce the potential $|\vec{u}|^4$, leaving the sign of this coefficient in the effective action indeterminate. However, we predict that the leading contributions to the $|\vec{u}\cdot\vec{u}|^2$ term are positive.

Resulting C_3 symmetry

Specifically, as pointed out in Ref. [7], the DSL on the triangular lattice admits a three-monopole term

$$S_\lambda = \lambda \int \mathrm{d}^3x\,[\Phi_1\Phi_2\Phi_3 + \text{h.c.}] \tag{10}$$

which is allowed by the microscopic (UV) symmetries (for example, this three-monopole term has zero lattice momentum since $\mathbf{X}_1+\mathbf{X}_2+\mathbf{X}_3=0$), but clearly breaks the emergent SO(6) symmetry at the QED_3 fixed point. Note that the three-monopole term has scaling dimension $\Delta_{\Phi\Phi\Phi}\approx 4.3$ in the large-N expansion and is therefore assumed to be irrelevant [1].

Considering the coupling of the DSL to displacement fields, any finite $\lambda\neq 0$ induces analogous corresponding anharmonic terms in the effective action,

$$\mathcal{E}_\lambda[\vec{u}] \sim \tilde{\lambda}\,[u_1u_2u_3 + \text{h.c.}]\,. \tag{11}$$

Within perturbation theory about QED_3, one contribution to the effective action of this form arises from $\langle S_g^3 S_\lambda\rangle/(\beta V)$. We stress that, since (11) is allowed by the microscopic symmetries of the system, it will also generically arise in any microscopic theory of lattice displacements upon going beyond the harmonic approximation, even in the absence of a coupling to the DSL (hence $\tilde{\lambda}$ is not necessarily related to λ in S_λ). We assume in the main text that this operator is dangerously irrelevant, such that instead of having a continuous SO(3) manifold of degenerate ground states, there are three degenerate ground states $\mathrm{e}^{2\pi\mathrm{i}n/3}\vec{u}_0$, with $n=0,1,2$. The resultant lattice distortion preserves the $C_3 = T_1C_6^2$ lattice symmetry and has an additional reflection symmetry. In real-space, the pattern is written

$$\mathbf{u}(\mathbf{r}) = 2\,\mathrm{Im}\left(\sum_a s_a\hat{\mathbf{X}}_a\,u_0^a\,\mathrm{e}^{\mathrm{i}\mathbf{X}_a\cdot\mathbf{r}}\right). \tag{12}$$

The spins react to the lattice breaking the translation and C_6 rotation by forming a valence bond solid state with the same symmetries, as described in the main text.

Supplementary Note 2: Kagome lattice

Our general framework and formalism can be straightforwardly applied to the U(1) DSL state on the Kagome lattice [9, 7]. On the Kagome lattice, the UV symmetry quantum numbers of the monopoles are different than those on the triangular lattice. In particular, the VBS monopoles carry lattice momenta $\mathbf{M}_a$, corresponding to the Brillouin zone edge centers. Further, under C_6 rotations, they pick up an additional phase: $s_a\Phi_a\to s_{a+1}\mathrm{e}^{-2\pi\mathrm{i}/3}\Phi_{a+1}$, and under reflection R the monopoles transform as Table 1 from the main text, but with an additional Hermitian conjugation [7]. The construction of the monopole-lattice coupling is complicated by the fact that the non-Bravais Kagome lattice has three sites per unit cells such that there are 6 (3 longitudinal + 3 transverse) distortion modes for a given lattice momentum.

Based on the previous analysis, we can focus on the longitudinal modes on the sublattice $X=A,B,C$ (an intra-unit cell index) and momenta $\mathbf{M}_a$ which we label as $u_X(\mathbf{M}_a)$. Under the symmetry operations, the different (real) Fourier components with wavevectors $\mathbf{M}_a$ are mapped onto each other (corresponding to changing monopole flavours). Simultaneously, the intra-unit cell coordinates $X=A,B,C$ are appropriately mapped into each other (this intra-unit cell contribution is similar to the 'pseudo-angular momentum' contribution of chiral phonons under C_6 as studied in Ref. [10]). Hence, the X components can be decomposed with respect to the irreducible representations (irreps) of $C_{3v}\simeq\mathcal{S}_3$ (the permutation group of three elements), which possesses three (complex) one-dimensional irreps which can be labeled by their C_3 eigenvalues. Choosing the appropriate irrep,

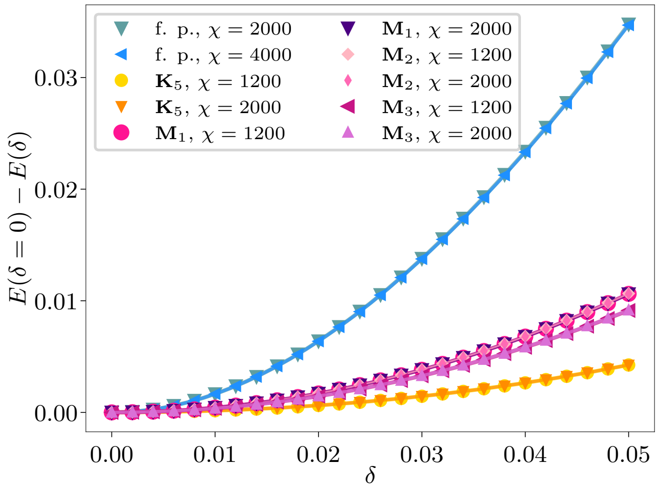

Supplementary Figure 1: Convergence of the DMRG simulations for different patterns as shown in Fig. 2a in the main text on an $L = 6$-cylinder. We show the energy gain for the full pattern (f. p.) as defined in Supplementary Eq. (12), for the three-sublattice pattern for $\mathbf{K}_5 = -\mathbf{K}_3$ with a phase of $\theta = \frac{1}{2}$ and for patterns associated to the midpoints $\mathbf{M}_i$ $(i = 1, 2, 3)$ of the edges of the Brillouin zone with phases $\theta_i = \frac{1}{6}, \frac{1}{2}, \frac{5}{6}$ respectively. Fig. 2a of the main text shows the average over the different orientations of the $\mathbf{M}$-point patterns. Calculations on the $L = 6$ cylinder have been performed by the flux-insertion method and a bond dimension of up to $\chi = 4000$; convergence is evident from the independence of the response on bond dimension. The distorted couplings in real space for the $\mathbf{K}$-pattern is shown in Supplementary Fig. 3a, for the $\mathbf{M}$-points in Supplementary Fig. 2c and Supplementary Fig. 3b–c.

h_a, that cancels out the additional phase factors of the monopoles under C_6 rotations, allows us to write the couplings

$$S_g[\vec{h}] \sim g \left(\mathrm{e}^{2\pi \mathrm{i}/3} u_1 \Phi_1 + u_2 \Phi_2 - \mathrm{e}^{-2\pi \mathrm{i}/3} u_3 \Phi_3 + \text{h.c.} \right). \tag{13}$$

Having obtained the coupling, the results from the main text carry over straightforwardly; the coupling precipitates an instability towards VBS ordering and lattice distortion. Transforming back to the sublattice basis $X = A, B, C$, the longitudinal phonon which couples to the monopoles is obtained by relative $2\pi/3$ phases $u_X(\mathbf{M}_a) = \mathrm{e}^{2\pi \mathrm{i}\, X/3} u_a$, allowing us to predict the lattice distortion as in Fig. 1 of the main text. Identifying symmetry-equivalent expressions of the monopole operators in terms of microscopic spin bilinears, we can additionally characterize the resulting ordered state on a microscopic level as the 'pinwheel' VBS order [11, 12].

Supplementary Note 3: Numerical Simulations

We use the density matrix renormalization group algorithm (DMRG) [13, 14, 15] on infinite cylinders [16, 17, 18] to obtain the ground state of the J_1–J_2 Heisenberg model on a triangular lattice. The lattice is wrapped onto the cylindrical geometry by closing the boundary conditions periodically along the circumference $L_y \equiv L$. There are several possible ways how the periodic closing of the lattice can be achieved. In this work, we use the $YCL_y - 0$ geometry [19, 20], where $n = 0$ determines the detailed boundary condition. The lattice sites $\mathbf{r}$ and $\mathbf{r} + L_y\mathbf{a}_2 - n\mathbf{a}_1$ are thereby identified. We use U(1)-charge conservation for all numerical simulations discussed in this work. We use the setup of infinite matrix product states (MPS) [14, 15] and optimise the ground state running infinite DMRG (iDMRG) on unit cells of size $L_y \times 3$, where $L_x = 3$ denotes the number of rings in the cylinder geometry.

When optimizing the ground state of the two-dimensional Hamiltonian on the lattice, special care has to be taken when we consider a geometry with an even circumference L_y. The isotropic $J_1 - J_2$ Heisenberg model on even cylinders comprises two different topological sectors in the putative quantum spin-liquid phase [21, 19]. The iDMRG algorithm in this case finds the even sector in which the entanglement spectrum on the bonds is symmetric around the total S_z-quantum number $q_z = 0$. One can transition into the odd sector by adiabatically inserting a flux of 2π through the cylinder [20]. In this process, the couplings of the model acquire complex

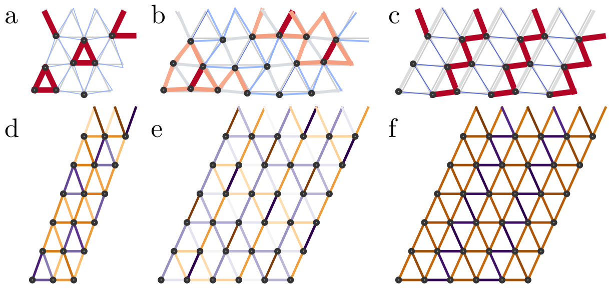

Supplementary Figure 2: Top subfigures **a**–**c** show the modified nearest-neighbour Heisenberg exchanges under the lattice distortion patterns $\mathbf{K}_3$, $-\mathbf{X}_3 \equiv \bar{\mathbf{X}}_3$ and $\mathbf{M}_3$ with the phases $\theta(\mathbf{K}_3) = \frac{1}{6}$, $\theta(\bar{\mathbf{X}}_3) = \frac{1}{6}$ and $\theta(\mathbf{M}_3) = \frac{5}{6}$. Bottom subfigures **d**–**f** show the respective nearest-neighbour ground state correlations $\langle \vec{S}_i \cdot \vec{S}_j \rangle$ for a distortion of $\delta = 0.0038$, with purple showing more negative VBS weight. For each subfigure **d**–**f**, the color scale has been normalised to the minimal and maximal value of the correlations measured. The patterns shown here have been used to study the finite-size effects as discussed in Fig. 2c–d of the main text.

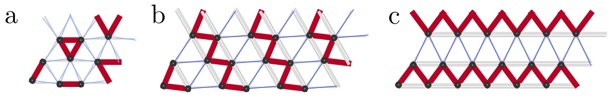

Supplementary Figure 3: Additional patterns as used for the DMRG simulations generated from the following parameters: **a** momentum $\mathbf{K}_5 \equiv -\mathbf{K}_3$ with phase $\theta = \frac{1}{2}$, **b** momentum $\mathbf{M}_1$ with phase $\theta = \frac{1}{6}$ and **c** momentum $\mathbf{M}_2$ with $\theta = \frac{1}{2}$. The corresponding energy responses under increasing δ are shown in Supplementary Fig. 1.

phases along the circumference. The final state's entanglement spectrum is symmetric around $q_z = \frac{1}{2}$ which can be understood as a spinon quasiparticle with $S_z = \pm\frac{1}{2}$ residing on each boundary [21]. To find the ground state for the $L_y = 6$-cylinder, we apply this protocol referred to as the flux-insertion method.

Alternatively, one can enforce a preference for odd dimer coverings on cylinders with even L_y by omitting a single site on each of the outermost cylinder rings of the unit cell for the first iDMRG sweeps before restoring the full model [19, 20] and having the algorithms optimise the ground state MPS until convergence. We refer to this protocol in contrast to the flux-insertion as the odd-sector method. We ensured that for sufficiently large bond dimensions, the energies obtained on $L_y = 6$ by the two distinct methods described above agree.

Starting from the ground state of the undistorted model, we can adiabatically turn on the distortion parameter δ and modify the couplings of the Hamiltonian according to the pattern generated by a certain momentum $\mathbf{Q}$. The parameter δ controls the strength of the distortion. The full 12-site unit cell pattern in real-space is obtained from the main text as follows:

$$\mathbf{r}_i = \mathbf{R}_i + \frac{2\delta}{\sqrt{3}} \sum_{a=1,2,3} s_a \hat{\mathbf{X}}_a \sin\left[\mathbf{X}_a \cdot \mathbf{r}_i + \frac{2\pi(a-1)}{3}\right], \tag{14}$$

where $\mathbf{R}_i$ denotes the undistorted position of site i and δ measures the magnitude of the distortion. All other patterns are generated via the formula

$$\mathbf{r}_i = \mathbf{R}_i + \delta \begin{pmatrix} \cos[\mathbf{Q} \cdot \mathbf{R}_i + \theta] \\ \sin[\mathbf{Q} \cdot \mathbf{R}_i + \theta] \end{pmatrix} \tag{15}$$

where the phase θ is an additional parameter that gives rise to the various patterns for each momentum $\mathbf{Q}$. The precise phases used for different distortion patterns are given in Supplementary Figs. 2 and 3 alongside the real-space modification of the couplings (for a value $\delta = 0.1$ for demonstration purposes).

For the investigation of the system size dependence, the simulated pattern at the commensurate monopole distortion for a single $\frac{\mathbf{K}}{2}$-momentum was taken as the negative $-\mathbf{X}_3 = \frac{\mathbf{K}_3}{2}$ with a phase of $\theta = \frac{1}{6}$. For the sake of readability, the corresponding data points in the figures in the main text have been named $\mathbf{X}_3$ (cf. Fig. 2 in

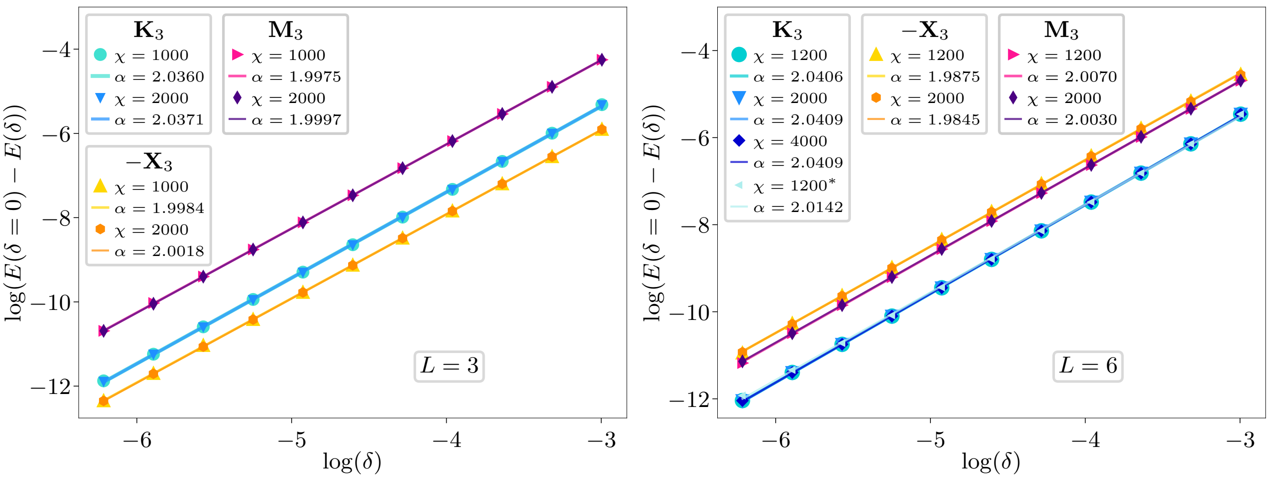

Supplementary Figure 4: Convergence of the DMRG simulations for $L = 3, 6$ and the three patterns generated by the momenta $\mathbf{K}_3$, $-\mathbf{X}_3$, and $\mathbf{M}_3$ for various bond dimensions. The real-space patterns are shown in Supplementary Fig. 2. For the $L = 6$ pattern generated by $\mathbf{K}_3$, the ground state was obtained by adiabatically inserting a flux of 2π through the cylinder, except for the curve marked with an asterisk; the odd-sector method was used in this case, and for all other patterns shown.

the main text). The pattern generated by $\mathbf{X}_3$ in contrast to $-\mathbf{X}_3$ would be mirrored along the $\mathbf{a}_2$-axis of the lattice, which is an irrelevant modification in terms of the physics of the system.

In the main text, we show the energy response for various patterns under adiabatic increase of the distortion δ. The $\mathbf{M}$-point patterns, as shown in Supplementary Figures 2c, and 3b–c, have different orientations on the cylinder with slightly differing energy responses. The main text therefore shows the averaged energy response over all three orientations. The shaded area indicates the range of the energy gain for the different orientations on the cylinder as shown explicitly in Supplementary Fig. 1.

To check convergence in the virtual bond dimension χ of the matrix product state, we can store the state along with the environments of the infinite DMRG simulation and restart the optimization under an increase of the bond dimension. The saving of the environments is necessary to ensure that the algorithm does not fall back into a possibly wrong topological sector.

In Supplementary Figures 1 and 4, we show the convergence for $L = 3$ and $L = 6$ for various patterns. The real-space distortions of the couplings alongside the spin-spin correlations $\langle \vec{S}_i \cdot \vec{S}_j \rangle$ resulting from the numerically obtained ground states are shown in Supplementary Fig. 2. As can be deduced from the figures, the energy difference is already extremely well-converged for intermediate bond dimensions of a few thousand. The absolute ground state energy, however, is not necessarily so well-converged for all circumferences and bond dimensions. Despite this, we have confidence in our results for the energy gain (even for small values $\mathcal{O}(10^{-10})$) because of the stricter convergence criteria applied to the DMRG algorithm.

Note that the size of the unit cell in x-direction, denoted by L_x, needs to be chosen in accordance with the pattern we simulate. More precisely, whereas the patterns for $\mathbf{K}$-points can be fitted on any multiple of three, i.e., $L_y \times 3$ is sufficient for the unit cell size to have a commensurate geometry (given that L_y itself is a multiple of three), the distortion patterns generated by the $\mathbf{M}$-points or $\frac{\mathbf{K}}{2}$ momenta, require an extent of $L_x = 6$ (or multiples thereof).

In contrast to $L_y = 3$ and $L_y = 6$, the circumference of $L_y = 9$ is more challenging as the computational complexity grows exponentially in L_y. In particular, the DMRG simulation tends to converge to semistable states that can change abruptly when bond dimension is insufficient. For small distortion strengths δ, the convergence is more stable for the accessible bond dimensions. In Supplementary Figure 5, we show data for various patterns for $L_y = 9$, which appear to converge for two of the three patterns studied for a bond dimension of $\chi = 6000$ and in the case of the $\mathbf{K}$-point for $\chi = 7000$.

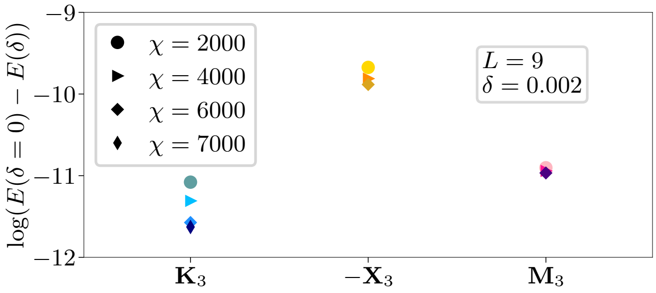

Supplementary Figure 5: Convergence of the DMRG simulations for $L = 9$ for a specific small distortion of $\delta = 0.002$ and the three patterns generated by the momenta $\mathbf{K}_3$, $-\mathbf{X}_3$ and $\mathbf{M}_3$ for various bond dimensions. The phases are chosen as defined in Supplementary Fig. 2. The circles denote data points for bond dimension $\chi = 2000$, the triangles for $\chi = 4000$, the diamonds for $\chi = 6000$ and the thin diamonds for $\chi = 7000$. Note the logarithmic scale on the y-axis.

Supplementary Note 4: Strong coupling approach

Generic scaling argument

Consider a general phase in $d = D + 1$ dimensions which has a conformally invariant ground state. Now, couple some relevant order parameter $\mathcal{O}$ to lattice distortions. In this general case, Eqs. (8) and (9) from the main text hold, with $\chi = d/(d - \Delta_{\mathcal{O}})$. This scaling analysis, and the leading perturbative result, find a spin-Peierls instability due to static lattice distortions when $\chi < 2$, or $\Delta_{\mathcal{O}} \leq d/2$, which occurs whenever fluctuations of the corresponding order are quantum-critical [22].

Lifting the accidental U(3) degeneracy

Note that the scaling ansatz for the strong-coupling regime possesses an U(3) symmetry, which is accidental given that the low-energy field theory only has a $\mathrm{SO}(3)_{\mathrm{valley}}$ degeneracy (see also Supplementary Note 1). To lift this accidental degeneracy, one could add a term of the form $\sim |\vec{u} \cdot \vec{u}|^{\chi/2}$ which transforms trivially under the field-theory's low-energy group, but explicitly breaks down U(3) $\to$ SO(3). However, at this stage, the sign of the coefficient of this term is left undetermined, such that the selected configuration may satisfy either $\hat{u} \cdot \hat{u} = 0$, or $\hat{u} \cdot \hat{u} = 1$ up to some phase (where $\hat{u} = \vec{u}/|\vec{u}|$). To fix the sign of this term (and thus select a particular configuration of $\hat{u}$), we may either appeal to consistency with perturbative approaches or use numerical methods to confirm it.

In the case of the DSL on the triangular lattice studied in the main text, the low-energy field theory description has an emergent IR symmetry group SO(6) with SO(3) spin and valley subgroups under which monopole operators (critical order parameters) transform as vectors. Similarly, VBS order parameters at deconfined quantum critical points transform under an emergent continuous symmetry group [e.g. SO(2)]. We note that by spin-rotation symmetry as well as lattice symmetries, VBS monopoles/order parameters can couple to lattice distortion modes u_a at appropriate momenta $\mathbf{Q}_a$, where $a = 1, \ldots, N$ labels N symmetry-equivalent momenta (valleys). In all examples discussed, N coincides with the order of the field theory's emergent SO(N) symmetry group. There is no reason to believe that the emergent symmetry is preserved when the system undergoes an instability/is driven away from the deconfined fixed point. Instead, only microscopic symmetries (e.g. spin rotations or lattice translation/rotations) are physical. Indeed in the case of the U(1) DSL, dangerously irrelevant operators (three-monopole terms), which are allowed by the microscopic symmetry group but break the emergent SO(6) symmetry, provide a mechanism for this.

CFT perspective on (in-)stability of free Dirac fermion systems

Finally, we take an instructive example of spin-Peierls instabilities by considering a simple conformal ground state: free fermions in one and two spatial dimensions. These emerge as low-energy theories for half-filled free fermions bands (in 1d), and graphene (in 2d). Fermion bilinears generally gap the spectrum, and have dimension $\Delta_M = d - 1$; hence we predict a spin-Peierls instability when $d \leq 1$. This is in agreement with

energetic calculations, in which one obtains a leading $-u^{3/2}$ energy gain for Kekule distortions in graphene (the Kekule lattice distortion mode couples to a fermion bilinear mass term), and a $-u^2\log(u)$ gain in 1D, where only logarithmic corrections lead to a weak instability. While this implies that a 2D system of free fermions does not exhibit a spin-Peierls instability and as such is more stable than the 1D free-fermion analogue, it is crucial to note that in strongly-interacting theories, there may exist many operators more relevant than fermion bilinears.

Supplementary Note 5: Correlation functions with dynamical phonons

Phase Transition

In the adiabatic limit $\omega_0 = 0$, the monopole-monopole interaction becomes non-local in time. This implies that modes become correlated at large (temporal) separations, leading to an ordering instability at infinitesimal couplings. Continuing to finite frequencies $\omega_0 > 0$, one may be tempted to perform a mean-field decoupling of the monopole-monopole interaction in $S_{\Phi\Phi}$. This would capture an ordering instability by some finite (and time-independent) monopole expectation value [6], $\Phi(x) \to \langle\Phi\rangle_{\rm MF}(x) \neq 0$. However, an explicit perturbative mean-field calculation (again employing an explicit IR regularization, e.g., by working at finite temperature) reveals a criticality condition identical to the static case, which crucially does not depend on ω_0. This is understood to be an artifact of mean-field theory which neglects the retardation of the monopole-monopole interaction in $S_{\Phi\Phi}$, and thus cannot capture any effects due to having a non-zero phonon frequency ω_0. This may also be seen explicitly: After decoupling, (schematic notation) $\Phi^\dagger\Phi \to \langle\Phi^\dagger\rangle_{\rm MF}\Phi + \Phi^\dagger\langle\Phi\rangle_{\rm MF} - \langle\Phi^\dagger\rangle_{\rm MF}\langle\Phi\rangle_{\rm MF}$, with time-independent $\langle\Phi\rangle_{\rm MF}$, the effective action becomes equivalent to the classical action $\mathcal{H}_{\rm ph} + \mathcal{H}_g[\mathbf{u}] + \mathcal{S}_{\rm QED_3}$, if one relates the monopole mean-field expectation value to an effective displacement field.

In the opposite, antiadiabatic, limit $\omega_0 \to \infty$ (with κ constant), the Green's function becomes purely local, $G(x-y) = (1/2\kappa)\,\delta^{(3)}(x-y)$. Note that in this limit, the monopole-monopole interaction becomes singular due to the OPE $\lim_{\epsilon->0}\Phi^\dagger(\epsilon+x)\Phi(x) \sim \mathbb{1}/\epsilon^{2\Delta_\Phi}$. To regularise this, one may work with a finite UV cutoff $\mathsf{a} \leq |x-y|$ that enforces a lower bound on the spatial monopole-monopole separation (given naturally by the lattice spacing). Then, upon taking $\omega_0 \to \infty$ at some fixed cutoff, the interaction effectively vanishes and the DSL remains stable. Considering both limits, it becomes clear that there must exist a transition at some intermediate coupling g determined by ω_0.

To obtain the critical interaction strength as a function of ω_0, we perform a scaling analysis using the exponential form of $G(\tau_x - \tau_y)$: For separations $|\tau_x - \tau_y| \gtrsim 1/\omega_0$, the interaction is exponentially suppressed, thereby setting a critical cutoff scale $\ell_c \sim 1/\omega_0$. Starting with some cutoff $\ell < \ell_c$, we can increase the cutoff up to ℓ_c where the interaction becomes suppressed. Rescaling all coordinates $x = x'/\ell_c$ (with x' now dimensionless) reveals the critical (dimensionless) interaction scale $1 \sim g^2\omega^{2\Delta_\Phi-3}/\kappa$, or equivalently $g_c^2 \sim \kappa\,\omega_0^{3-2\Delta}$ (where we use that $|\mathbf{k}| \sim 1/\mathsf{a}$ with a being the lattice spacing).

Critical Coupling

We now investigate the stability of the DSL in the presence of the interaction $S_{\Phi\Phi}$ within perturbation theory. Considering a perturbative expansion of local observables, the expansion's breakdown is taken to signal an instability of the DSL. We employ the path-integral formalism, where the expectation value of some observable $\mathcal{X}$ is written as $\langle\mathcal{X}\rangle = \mathcal{Z}^{-1}\int\mathcal{D}[\{\mathcal{O}_{\rm CFT}\}]\,\mathcal{X}\mathrm{e}^{-S}$ where S is the system's interacting action and $\mathcal{Z}$ its partition function without operator insertion, as above.

At the QED$_3$ fixed point (i.e., zeroth order perturbation theory), all one-point functions vanish at zero temperature $\beta \to \infty$ in the thermodynamic limit by conformal symmetry. We thus turn to the monopole two-point function at large distances $|d| = |(\tau_d, \mathbf{r}_d)| \to \infty$. Expanding the Boltzmann weight $\mathrm{e}^{-S_{\Phi\Phi}}$ in the path integral to first order, one finds the perturbative expansion

$$\langle\Phi_a^\dagger(d)\Phi_b(0)\rangle_{g^2} = \frac{\delta_{ab}}{|d|^{2\Delta_\Phi}} - \left(\langle\Phi_a^\dagger(d)\Phi_b(0)S_{\Phi\Phi}\rangle_{\rm QED_3} - \frac{\delta_{ab}}{|d|^{2\Delta_\Phi}}\langle S_{\Phi\Phi}\rangle_{\rm QED_3}\right) + \dots, \tag{16}$$

where we have used the two-point functions evaluated at the CFT fixed point. The first term in the parenthesis

is written explicitly as

$$\langle \Phi_a^\dagger(d)\Phi_b(0)S_{\Phi\Phi}\rangle_{\mathrm{QED}_3} = -\sum_c \frac{g^2|\mathbf{X}_c|^2}{2\rho\omega_0}\int \mathrm{d}^3x\mathrm{d}^3y\left[\delta^{(2)}(\mathbf{r}_x-\mathbf{r}_y)\mathrm{e}^{-\omega_0|\tau_x-\tau_y|}\langle \Phi_a^\dagger(d)\Phi_b(0)\Phi_c^\dagger(x)\Phi_c(y)\rangle_{\mathrm{QED}_3}\right]. \tag{17}$$

Note that while 2- and 3-pt. functions of primaries are exactly determined by conformal symmetry, no closed forms are available for 4-pt. functions, making the evaluation of Supplementary Eq. (16) challenging.

While Wick's theorem does not hold in interacting CFTs, we can successively apply operator product expansions (OPE) to decompose the 4-pt. function in (17). If the full (convergent) OPE is used, the choice of OPE channels is inconsequential (indeed, demanding OPE associativity lies at the heart of CFT bootstrap studies [23]). However, for analytical tractability, we will use the asymptotic forms of OPE [see also Supplementary Eq. (2)]. A particular OPE channel then corresponds to a particular configuration of operator insertions which yield the most divergent contribution in the particular channel if they become 'close'.

Considering Supplementary Eq. (17), we will focus on the leading monopole-antimonopole OPE channel (schematically $\Phi^\dagger \times \Phi = \mathbb{1}$) corresponding to $x \to 0$ and $y \to d$, with x and y sufficiently far apart, which means that we may assume $0 < |x| \ll |y|$. Explicitly, the first-order OPE in this channel then reads

$$\langle \Phi_a^\dagger(d)\Phi_b(0)\Phi_c^\dagger(x)\Phi_c(y)\rangle_{\mathrm{QED}_3} \sim \delta_{ac}\delta_{bc}\frac{1}{|d-y|^{2\Delta_\Phi}|x|^{2\Delta_\Phi}}. \tag{18}$$

We expect this channel to give the most dominant contribution: The other monopole-antimonopole channel corresponding to $d \to 0$, $x \to y$ is asymptotically equivalent to the disconnected contribution (second term in the parenthesis) in Supplementary Eq. (17), and is thus expected to only weakly contribute to $\langle \Phi_a^\dagger(d)\Phi_b(0)\rangle_{g^2}$.

We use (18) in (17). As $|d| \to \infty$, we can take $|d-y|^{-2\Delta_\Phi} \approx |d|^{-2\Delta_\Phi}$ and subsequently perform the $\mathbf{r}_y$-integration, yielding

$$\langle \Phi_a^\dagger(d)\Phi_b(0)S_{\Phi\Phi}\rangle_{\mathrm{QED}_3} \approx -\frac{\delta_{ab}}{|d|^{2\Delta_\Phi}}\frac{g^2|\mathbf{X}_a|^2}{2\rho\omega_0}\int \mathrm{d}^3x\int \mathrm{d}\tau_y\,\frac{\mathrm{e}^{-\omega_0|\tau_x-\tau_y|}}{(|\mathbf{r}_x|^2+\tau_x^2)^{\Delta_\Phi}}. \tag{19}$$

We work on a cylindrical geometry $S^1_\beta \times \mathbb{R}^2 \overset{\beta\to\infty}{\to} \mathbb{R}^3$. The spatial integration is performed in polar coordinates with $0 < |\mathbf{r}_x| < L \to \infty$,

$$|d|^{2\Delta_\Phi}\langle \Phi_a^\dagger(d)\Phi_b(0)S_{\Phi\Phi}\rangle_{\mathrm{QED}_3} \approx -\delta_{ab}\frac{\pi g^2|\mathbf{X}_a|^2}{2(\Delta_\Phi-1)\rho\omega_0}\int \mathrm{d}\tau_x\mathrm{d}\tau_y\,\frac{\mathrm{e}^{-\omega_0|\tau_x-\tau_y|}}{|\tau_x|^{2\Delta_\Phi-2}}. \tag{20}$$

Note that due to the δ-distribution for the spatial components in (17), only configurations with $\mathbf{r}_x = \mathbf{r}_y$ give a finite contribution. Thus, our previous assumption of $|x| \ll |y|$ implies for the temporal coordinates $|\tau_x| \ll |\tau_y|$. With $|\tau_x - \tau_y| \approx |\tau_y|$, the remaining integrals are obtained as

$$\lim_{\beta\to\infty}\int_0^\beta \mathrm{d}\tau_y\int_0^{|\tau_y|}\mathrm{d}\tau_x\,\frac{\mathrm{e}^{-\omega_0|\tau_y|}}{|\tau_x|^{2\Delta_\Phi-2}} \approx \frac{1}{3-2\Delta_\Phi}\frac{\Gamma(4-2\Delta_\Phi)}{\omega_0^{4-2\Delta_\Phi}}, \tag{21}$$

which exists for $\Delta_\Phi < 2$, which is within the range of assumed scaling dimensions. Hence, one finds

$$|d|^{2\Delta_\Phi}\langle \Phi_a^\dagger(d)\Phi_b(0)S_{\Phi\Phi}\rangle_{\mathrm{QED}_3} \approx -\delta_{ab}\frac{c_{\Delta_\Phi}\Gamma(4-2\Delta_\Phi)}{4\rho\omega_0^2}\frac{g^2|\mathbf{X}_a|^2}{\omega_0^{3-2\Delta_\Phi}} \tag{22}$$

Generically, perturbation theory breaks down when the $(n+1)$-term in the expansion is no longer small compared to the n-th term. Here, we compare the first-order correction to the bare correlator,

$$\frac{\delta^{(1)}\langle \Phi_a^\dagger(d)\Phi_a(0)\rangle_{g^2}}{\langle \Phi_a^\dagger(d)\Phi_a(0)\rangle_{\mathrm{QED}_3}} \approx \frac{1}{4}c_{\Delta_\Phi}\Gamma(4-2\Delta_\Phi)\frac{g^2|\mathbf{X}_a|^2}{\mathcal{K}_{\mathbf{X}_a}\omega_0^{3-2\Delta_\Phi}}. \tag{23}$$

Perturbation theory breaks down if this ratio is of order 1. This leads to a scaling relation for the critical coupling, given by

$$g_c^2/\kappa \sim \omega_0^{3-2\Delta_\Phi}. \tag{24}$$

In the thermodynamic limit at zero temperature, the finite phonon frequency $\omega_0 \neq 0$ hence prevents a weak-coupling instability and rather determines a critical g_c as a function of ω_0. The critical g_c matches precisely the result of the scaling analysis in the main text.

Supplementary References

[1] Shai M. Chester and Silviu S. Pufu. Towards bootstrapping qed3. *Journal of High Energy Physics*, 2016(8):19, 2016.

[2] Soner Albayrak, Rajeev S. Erramilli, Zhijin Li, David Poland, and Yuan Xin. Bootstrapping $N_f = 4$ conformal qed$_3$. *Phys. Rev. D*, 105:085008, Apr 2022.

[3] Thomas Appelquist, Daniel Nash, and L. C. R. Wijewardhana. Critical behavior in (2+1)-dimensional qed. *Phys. Rev. Lett.*, 60:2575–2578, Jun 1988.

[4] Tarun Grover. Entanglement monotonicity and the stability of gauge theories in three spacetime dimensions. *Phys. Rev. Lett.*, 112:151601, Apr 2014.

[5] Jens Braun, Holger Gies, Lukas Janssen, and Dietrich Roscher. Phase structure of many-flavor qed$_3$. *Phys. Rev. D*, 90:036002, Aug 2014.

[6] Zhu-Xi Luo, Urban F. P. Seifert, and Leon Balents. Twisted bilayer u(1) dirac spin liquids. *Phys. Rev. B*, 106:144437, Oct 2022.

[7] Xue-Yang Song, Chong Wang, Ashvin Vishwanath, and Yin-Chen He. Unifying description of competing orders in two-dimensional quantum magnets. *Nature Communications*, 10(1):4254, 2019.

[8] Xue-Yang Song, Yin-Chen He, Ashvin Vishwanath, and Chong Wang. From spinon band topology to the symmetry quantum numbers of monopoles in dirac spin liquids. *Phys. Rev. X*, 10:011033, Feb 2020.

[9] Ying Ran, Michael Hermele, Patrick A. Lee, and Xiao-Gang Wen. Projected-wave-function study of the spin-1/2 heisenberg model on the kagomé lattice. *Phys. Rev. Lett.*, 98:117205, Mar 2007.

[10] Hao Chen, Weikang Wu, Shengyuan A. Yang, Xiao Li, and Lifa Zhang. Chiral phonons in kagome lattices. *Phys. Rev. B*, 100:094303, Sep 2019.

[11] A. V. Syromyatnikov and S. V. Maleyev. Hidden long-range order in kagomé heisenberg antiferromagnets. *Phys. Rev. B*, 66:132408, Oct 2002.

[12] K. Matan, T. Ono, Y. Fukumoto, T. J. Sato, J. Yamaura, M. Yano, K. Morita, and H. Tanaka. Pinwheel valence-bond solid and triplet excitations in the two-dimensional deformed kagome lattice. *Nature Physics*, 6(11):865–869, 2010.

[13] Steven R. White. Density matrix formulation for quantum renormalization groups. *Phys. Rev. Lett.*, 69:2863–2866, Nov 1992.

[14] Ulrich Schollwöck. The density-matrix renormalization group in the age of matrix product states. *Ann. Phys.*, 326:96 – 192, 2011.

[15] Johannes Hauschild and Frank Pollmann. Efficient numerical simulations with Tensor Networks: Tensor Network Python (TeNPy). *SciPost Phys. Lect. Notes*, page 5, 2018.

[16] Ian P McCulloch. Infinite size density matrix renormalization group, revisited. *arXiv:0804.2509*, 2008.

[17] E.M. Stoudenmire and Steven R. White. Studying two-dimensional systems with the density matrix renormalization group. *Annual Review of Condensed Matter Physics*, 3(1):111–128, 2012.

[18] Matthias Gohlke, Ruben Verresen, Roderich Moessner, and Frank Pollmann. Dynamics of the kitaev-heisenberg model. *Phys. Rev. Lett.*, 119:157203, Oct 2017.

[19] Zhenyue Zhu and Steven R. White. Spin liquid phase of the $s = \frac{1}{2}$ $J_1 - J_2$ heisenberg model on the triangular lattice. *Phys. Rev. B*, 92:041105, Jul 2015.

[20] Shijie Hu, W. Zhu, Sebastian Eggert, and Yin-Chen He. Dirac spin liquid on the spin-1/2 triangular heisenberg antiferromagnet. *Phys. Rev. Lett.*, 123:207203, Nov 2019.

[21] Wen-Jun Hu, Shou-Shu Gong, Wei Zhu, and D. N. Sheng. Competing spin-liquid states in the spin-$\frac{1}{2}$ heisenberg model on the triangular lattice. *Phys. Rev. B*, 92:140403, Oct 2015.

[22] M. Zacharias, A. Rosch, and M. Garst. Critical elasticity at zero and finite temperature. *The European Physical Journal Special Topics*, 224(6):1021–1040, 2015.

[23] David Poland, Slava Rychkov, and Alessandro Vichi. The conformal bootstrap: Theory, numerical techniques, and applications. *Rev. Mod. Phys.*, 91:015002, Jan 2019.
